# A Capacitive Immunosensor Based on a Polypyrrole–CTAB for Probe-Free Detection of SARS-CoV-2 Spike Protein

**DOI:** 10.3390/mi17060731

**Published:** 2026-06-17

**Authors:** Licia de S. Gonçalves, Jose M. V. Fonseca, Nayara da S. Melo, Yonny Romaguera-Barcelay, Rosa F. Dutra

**Affiliations:** 1 Department of Biomedical Engineering, Federal University of Pernambuco, Recife 50670-901, Pernambuco, Brazil; 2 Cabo de Santo Agostinho Unit, Federal Rural University of Pernambuco, Cabo de Santo Agostinho 54518-430, Pernambuco, Brazil; 3 National Institute of Science and Technology in Bioanalytic—Lauro Kubota, Campinas State University, Campinas 13083-970, São Paulo, Brazil

**Keywords:** COVID-19, capacitive biosensor, polypyrrole, point-of-care

## Abstract

A capacitive screen-printed electrode immunosensor operating in non-faradaic mode by dispensing redox probes was developed for the Coronavirus 2 Spike (S) protein. This new strategy enabled direct detection of the S protein by measuring changes in the electrochemical capacitance resulting from antigen–antibody interactions on the electrode surface, altering interfacial dielectric properties. To enhance analytical sensitivity and provide an electrode surface with attractive capacitive and conductive properties, an in-house graphite ink-based screen-printed electrode was developed and subsequently modified with a polypyrrole (PPy) layer in bulk-synthesized in the presence of Cetyltrimethylammonium bromide (CTAB). CTAB acted as a dispersing and structure-directing agent, promoting homogeneous distribution and guiding the PPy polymerization, resulting in a composite with improved charge density storage and high conductivity. Analytical signals of the S proteins in spiked serum were detected by measuring the Specific Capacitances taken from cyclic voltammograms. This capacitive immunosensor achieved a linear range from 1 to 100 µg/mL (R^2^ = 0.989, *p* < 0.05), with a limit of detection of 0.45 µg/mL of S protein, which falls within the clinical range for COVID-19 diagnostics. Probe-free detection without ferri/ferrocyanide steps minimizes errors by probe adsorptions and is easy to use as a point-of-care, unlike conventional immunosensors.

## 1. Introduction

The coronavirus disease 2019 (COVID-19) caused the severe acute respiratory syndrome coronavirus 2 (SARS-CoV-2), emerged in late 2019 and rapidly escalated into a global public health crisis [1,2,3]. Declared a pandemic by the World Health Organization (WHO) in March 2020, COVID-19 has affected more than 230 countries and territories, with over 770 million confirmed cases and more than 7 million deaths reported worldwide [4]. Importantly, excess mortality estimates indicate that the actual global death toll may have reached approximately 14.9 million between 2020 and 2021, reflecting both direct viral effects and indirect consequences associated with healthcare system disruptions [1]. The unprecedented scale and rapid spread of SARS-CoV-2 have exposed critical limitations in conventional diagnostic strategies. Although vaccines have been developed, the rapid spread of the disease and the constant mutation of the virus denote the possibility of new outbreaks [5]. Even though molecular tests based on RNA amplification, such as real-time RT-PCR (RT-qPCR), CRISPR-based tests, and RT-Lamp have been used because they are a precise and sensitive technology and considered the gold standard [6,7], they require a complex laboratory infrastructure, longer processing time, and rigorous care in sample handling, representing a high risk of cross-reactions when applied on a large scale [8]. These requirements during the pandemic have made it evident that the molecular technique of RT-PCR did not exhibit enough robustness to be used on a massive scale, which could lead to the collapse of the health services [9]. Driven by the urgent demand for rapid and decentralized diagnostics, lateral flow tests targeting the Spike (S) protein have become a key alternative to conventional molecular methods.

Protein S is one of the main biomarkers for diagnostics, vaccines and therapies of SARS-CoV-2, since its presence indicates exposure to the virus [10]. Protein S detection allows identifying directly and specifically the infection, because it mediates the binding of the virus to host cells and facilitates its entry, being responsible for the recognition of specific cell receptors [11,12]. However, lateral flow tests suffered from reduced sensitivity, particularly during early stages of infection, when antigen or antibody levels are below detectable thresholds [13]. These limitations reinforce the urgent need for alternative diagnostic methods that enable rapid, accurate, and decentralized detection since there is always the possibility of new outbreaks of the disease, as new variants emerge all the time and the disease spreads rapidly [11,12].

Electrochemical immunosensors based on screen-printed electrodes (SPE) have been shown as an attractive alternative since, besides the advantages mentioned, they allow detections with high sensitivity, are scalable, and can be massively used [14]. In this context, numerous screen-printed electrode-based immunosensors have been developed using analytical techniques such as impedance spectroscopy, square wave voltammetry, and differential pulse voltammetry [15]. Considering that the antigen–antibody interaction does not generate electroactive species, unlike oxidoreductase enzymes, the analytical signals are mediated by the difference in the transfer rate of diffusional charges that act as a function of the diffusion barrier that becomes increased due to the antigens, of an insulating nature, adsorbing to the electrode surface by the specific interaction with the immobilized antibodies [16]. Thus, analytical detection is mediated by increasing the resistance of charge transfer (Rct) or by reducing the diffusional current that can be monitored using voltammetric techniques [17]. Although great progress has been achieved, these biosensors require redox probes or electroactive species to generate analytical signals. However, the presence of such probes as potassium ferri/ferrocyanide can absorb on the sensor surface, resulting in electrode poisoning, alteration of the baseline, loss of immunological activity, and false responses [18,19]. Probe-free systems with redox probes immobilized on the electrode surface have been proposed to overcome these inconveniences, offering several advantages such as being faster and more practical [19,20,21]. However, many challenges persist, such as controlling the baseline and maintaining the electroactive species on the electrode surface for the long term.

One way to overcome these limitations is the non-use of redox probes for the generation of the analytical responses [20,21,22]. In non-faradaic mode, the Imaginary component of Impedance related to double-layer capacitance is evidenced and preponderant, while the diffusional current is suppressed, reducing the inconveniences previously mentioned. Herein, it was possible to observe changes in electrochemical capacitance due to antigen–antibody interactions. A strategy to improve signal-to-noise relationships and to increase sensitivity is to obtain an electrode surface with a high electrochemical capacitance [23]. Polypyrrole (PPy) is one of the most widely studied conductive polymers due to its high chemical stability, good conductivity and ease of synthesis by polymerization both in-bulk, as well as in situ, being used in different capacitive devices and sensors [24,25,26]. Futhermore, PPy-based materials allow supercapacitive characteristics, combining high capacitance, fast charge–discharge rates, and good electrochemical reversibility [27,28]. The incorporation of surfactants such as cetyltrimethylammonium bromide (CTAB) during polymerization enables control over the morphology of PPy, improves dispersion, and promotes the formation of a well-defined functional nanostructure [29,30,31,32]. The use of carbon-based materials can further enhance charge-storage capacity. Combined with electrochemical characteristics, materials such as graphite [33], carbon nanotubes [30], and others have allowed the construction of affordable and highly conductive SPEs [34]. Graphite has been widely exploited in the construction of electrochemical transducers mainly due to its low cost. Its conductivity and stability can be especially improved when subjected to physical or chemical treatments that increase surface area and electrochemical reactivity [35]. Herein, the combination of PPy and graphite in the sensor surface forms robust electroactive composites capable of producing high capacitance, playing a central role in the electrochemical performance of the system, and favoring the predominance of the electrical double layer capacitance as the main contribution to the analytical signal. This behavior is associated with an increase in the effective surface area and high density of charges stored at the electrode/electrolyte interface, which amplifies capacitive variations resulting from molecular recognition events, such as antigen–antibody interactions. In addition, the high capacitance of the composite contributes to minimizing the influence of diffusion processes on electrochemical response. In conventional systems, the diffusion current, associated with the transport of redox species in the solution, can mask or interfere with the detection of small interfacial variations. However, in the proposed composite, the response is dominated by non-faradaic phenomena, significantly reducing the dependence on mass transport processes. Consequently, the system allows sensitive and reproducible measurements to be made without the need for redox probes in solution, avoiding problems such as nonspecific adsorption, destabilization of biomolecules and chemical interferences. This approach simplifies the experimental protocol and makes the sensor more suitable for applications in complex samples, increasing its robustness and analytical specificity [24,34].

In this study, anti-Spike antibodies were immobilized onto the composite-modified electrode surface, and the analytical response was obtained from variations in electrochemical capacitance induced by antigen–antibody interactions at the interface. These interactions modulate the interfacial properties of the electrode, leading to measurable capacitance changes. To the best of our knowledge, this is the first report demonstrating the direct detection of the S protein using a probe-free electrochemical capacitive immunosensor, eliminating the need for redox mediators and enabling a simplified strategy that is more suitable for large-scale or epidemiological scenarios.

## 2. Experimental

### 2.1. Reagents and Materials

Pyrrole (Py, 98%), N,N-dimethylformamide (DMF) and SunTronic^®^ conductive graphite ink for flexographic printing were obtained from Sigma-Aldrich (St. Louis, MO, USA). Graphite powder was purchased from Fisher Chemical (Waltham, MA, USA). Potassium hexacyanoferrate (II) trihydrate (K_4_[Fe(CN)_6_]3H_2_O) and tris(hydroxymethyl)aminomethane (TRIS) were obtained from Vetec (São Paulo, SP, Brazil). Potassium hexacyanoferrate(III) (K_3_[Fe(CN)_6_]), potassium nitrate (KNO_3_), potassium chloride (KCl), and phosphate-buffered saline (PBS) were purchased from Neon (São Paulo, SP, Brazil). All chemicals used in this study were of analytical grade, and ultrapure water was obtained from a Milli-Q purification system (Millipore, Bedford, MA, USA).

The anti-Spike polyclonal antibody produced in rabbits was acquired from RheaBiotech (Campinas, SP, Brazil). The Spike (S) protein was produced at the Laboratory of Cell Culture Engineering, COPPE, Federal University of Rio de Janeiro (UFRJ), Rio de Janeiro, Brazil. Serum samples used for S protein spiking were acquired from Sigma-Aldrich H4522 (St. Louis, MO, USA).

Human blood samples were collected by venipuncture from five healthy volunteers who tested negative for COVID-19 and were used to prepare pooled serum for analytical evaluation. Spiked samples were obtained by diluting the pooled serum in 50% (*v*/*v*) PBS, followed by the addition of defined concentrations of SARS-CoV-2 S protein while maintaining constant volumes to minimize matrix effects.

All procedures were conducted in accordance with the Declaration of Helsinki and approved by the Research Ethics Committee of the Aggeu Magalhães Institute (Fiocruz), Recife, Brazil (protocol no. 63441516.6.0000.5190).

### 2.2. Apparatus and Electrochemical Measurements

Electrochemical measurements were performed using an eight-channel potentiostat (Metrohm DropSens, Oviedo, Spain), interfaced with a computer and controlled via DropView 8400 Software (Metrohm DropSens, Oviedo, Spain). Home-made screen-printed carbon electrodes (SPEs) were fabricated by screen-printing a carbon ink formulation to define the working, counter, and reference electrodes. The working electrode (3 mm diameter) enabled low-volume analyses. Measurements were carried out at room temperature (~24 °C) by depositing 25 μL of sample onto the electrode surface, connected to the potentiostat through a dedicated DSC cable connector. For comparison, commercial SPEs (DRP-C110, Metrohm DropSens, Oviedo, Spain) were also evaluated.

Analytical responses were obtained by cyclic voltammetry (CV) at 50 mV/s scan rate, using PBS containing 0.5% Tween 20 as the supporting electrolyte. Likewise, to characterize the sensor platform, CV profiles were performed in the presence of 5 mM of K_3_Fe(CN)_6_/K_4_Fe(CN)_6_ prepared in 0.1 M KCl as redox probe. To measure the double layer effects and pseudocapacitance, supporting electrolyte as well as PBS was used instead of redox probe as mentioned.

Electrochemical impedance spectroscopy (EIS) was additionally used to characterize the stepwise modification of the sensor surface. Impedance spectra were recorded in 5 mM K_3_[Fe(CN)_6_]/K_4_[Fe(CN)_6_] prepared in 0.1 M KCl over the frequency range from 100 kHz to 0.1 Hz, using an AC amplitude of 40 mV. These measurements were performed only for interfacial characterization of the electrode modification steps. All EIS measurements were carried out inside a Faraday cage to minimize external electrical noise and improve signal stability.

### 2.3. Fabricating of the In-House SPE

The SPEs were fabricated by screen-printing a carbon paste, performed by a composite of graphite and carbon ink onto a PET substrate. This carbon paste was prepared by dispersing graphite powder (<20 μm) in DMF for 2 h in an ultrasonic bath at 45 °C and mixed with a SunTronic^®^ commercial flexographic conductive ink. Then, this carbon paste prepared with graphite and SunTronic^®^ carbon ink was homogenized in a mechanical stirrer until a viscosity of 1.3 to 1.7 Pa·s was obtained.

SPE was manufactured by squeezing the prepared carbon paste onto a vinyl adhesive printing, a mold mask, which was bonded to a rigid polyethylterephthalate (PET) substrate. This carbon composite was applied with a putty knife until 3 layers of carbon paste were reached; each layer was cured at 27 °C for 30 min in an oven. This sequential layering and curing approach was essential for forming a consistent, robust film in screen-printed carbon electrode fabrication. After curing the SPEs, the mold mask was removed, and the fabricated SPEs were then cut from the printed PET sheet. The electrode areas were delineated by a layer of nail polish on the conductive track. Before PPy composite modifications, the SPEs were polished with 2500-grit sandpaper and chemically treated with 0.5 M H_2_SO_4_ for 10 min.

### 2.4. PPy-CTAB Modifications on the SPE

PPy composite was synthesized by dispersing CTAB in 3 mL of deionized water under sonication for 1 h, preserving a pyrrole-to-CTAB molar ratio of 4:1. Then, 0.2 mL of pyrrole and 0.5 mL of 1.0 M H_2_SO_4_ were added, and the solution was stirred for 1 h in an ice-water bath. The resulting mixture of Py and CTAB was then added dropwise to a 1.0 M ammonium persulfate solution (APS), maintaining an oxidant-to-pyrrole molar ratio of 1:1, under vigorous stirring in an ice bath. The reaction was allowed to proceed for 4 h under cooling conditions.

Before SPE modification, the PPy composite obtained was washed three times with distilled water by centrifugation (5000 rpm for 16 min), filtered, and dried in an oven at 37 °C for 12 h. Afterwards, PPy powder obtained was dispersed in DMF, and 5 µL was pipetted onto the working electrode SPE surface, and incubated at 37 °C for 40 min in the dark. This procedure was repeated three times to obtain a three-layer PPy-CTAB composite SPE.

### 2.5. Anti-Spike Immobilization and Analytical Responses

The anti-Spike antibodies were immobilized on modified PPy-CTAB/SPE by pipetting one aliquot (3 µL) of anti-Spike antibody solution in a concentration of 50 µg/mL prepared in PBS (pH 7.4) onto the working electrode surface, which was incubated overnight in a refrigerator (4 to 6 °C). Afterwards, the excess unbound antibodies were removed by two washes, with the electrode immersed in PBS (pH 7.4) and gently stirred for 30 s during each wash. Then, to block non-specific binding, the electrode surface was incubated for 120 min at room temperature (approximately 23 °C) with a BSA solution (0.5% *w*/*v*) prepared in PBS. Finally, excess unbound BSA proteins were removed by 3 washes in PBS. All the incubation steps were performed by subjecting the working electrode to a moist chamber, avoiding drying and protecting the microenvironment from injuries.

PBS containing 0.5% (*v*/*v*) Tween-20 was used in the analytical measurements to maintain adequate wetting of the immunoelectrode interface and to limit nonspecific protein adsorption on the carbon–polymer surface. This condition favors a more reproducible biomolecular interface during antigen incubation, and subsequent capacitance measurements without contributing as a redox mediator or electroactive species [36,37].

Analytical measurements were performed by exposing the Anti-Spike-SPE in a solution containing the Spike antigen for 20 min in a humid chamber. Before measurements, the immunoelectrode was washed with PBS, and connected to the potentiostat, and an illustrative scheme of all preparation steps are exhibited in Supplementary Figure S1.

### 2.6. Raman Study and Morphological Characterization

Raman spectra were collected using a XploRA™ Raman Microscope (HORIBA Scientific, Kyoto, Japan) with a 532 nm laser (10 mW) to minimize sample heating. Spectra were recorded from 50 to 3600 cm^−1^ with a spectral resolution of 2 cm^−1^.

Surface morphology was analyzed by scanning electron microscopy (SEM) using a TESCAN VEGA3 microscope (TESCAN, Brno, Czech Republic) operated at 15 kV and coupled to Energy-Dispersive Spectroscopy (EDS) for elemental analysis.

## 3. Results and Discussion

### 3.1. SPE Manufacturing and Graphite-PPy Composite

#### 3.1.1. Raman Characterization

Raman spectroscopy was employed to structurally analyze compounds present in the home-made graphite carbon paste to prepare the screen-printed electrode, and after it has been modified by a capacitive film formed by PPy synthetized in bulk through APS and CTAB by chemical oxidation in an acidic medium.

Morphological characterizations through SEM micrography were performed on the electrode surfaces to evaluate the effects of polishing with 2500 grit sandpaper and, afterwards, chemical treatment with H_2_SO_4_ solution to remove electrochemical impurities on the SPE surface. The contributions of each component of the immunoelectrode were systematically investigated. A control SPE was first fabricated by screen-printing using only the commercial SunTronic^®^ conductive graphite ink without any modifications. Subsequently, SPEs were prepared from a carbon paste consisting of a SunTronic^®^/graphite powder composite. The role of CTAB was then evaluated by comparing PPy films synthesized in the presence and absence of the surfactant. Finally, selected electrodes were subjected to mechanical polishing with 2500-grit sandpaper, followed by chemical cleaning using 0.5 M H_2_SO_4_.

**Figure 1:**
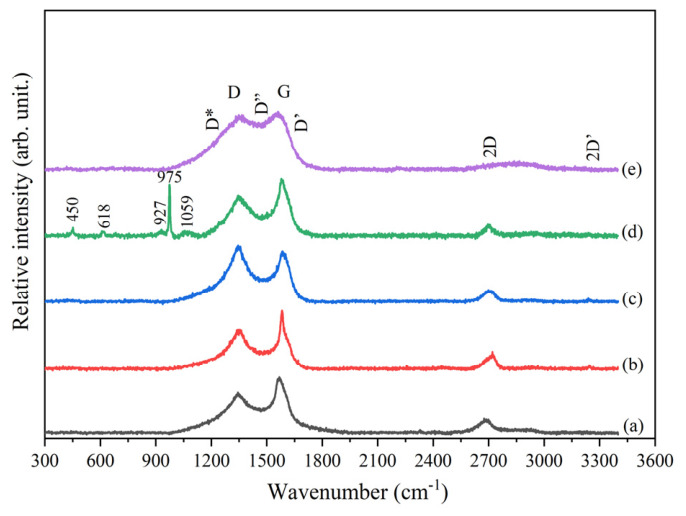
Raman spectra of SPE prepared with (a) SunTronic^®^ ink, (b) graphite–SunTronic^®^, (c) polished/acid-treated graphite–SunTronic^®^, (d) polished/acid-treated graphite–PPy (without CTAB), and (e) polished/acid-treated graphite–PPy (with CTAB).

In Figure 1 the Raman spectra of SPE is formed by: (a) SunTronic^®^, (b) graphite–SunTronic^®^, (c) polished/acid-treated graphite–SunTronic^®^, (d) polished/acid-treated graphite–PPy (without CTAB), and (e) polished/acid-treated graphite–PPy (with CTAB). The main Raman band positions and assignments are summarized in Table 1, while the intensity ratios and crystallite size estimates are reported in Table 2. All spectra presented in Figure 1 exhibited the characteristic Raman fingerprint of sp^2^ carbon materials, dominated by the D band (~1347 to 1361 cm^−1^) and the G band (~1555 to 1584 cm^−1^), as listed in Table 1. These bands are widely used to describe disorder and graphitic domains in graphite- and graphene-based electrodes [33,38,39]. In addition, all samples show a second-order 2D band in the ~2683 to 2728 cm^−1^ range, together with a combination band around ~2888 to 2956 cm^−1^, which are typical of graphene-related carbons and sensitive to structural organization [40,41]. Figure 1(a),(b) show that SPE-COM and SPE-FAB have almost identical spectra, with similar band locations and relative intensities, indicating equivalent carbon structures.

The similarity as observed in Figure 1 is confirmed by the quantitative analysis reported in Table 2. The I_D_/I_G_ ratios obtained for SPE-COM (0.70) and SPE-FAB (0.78) fall within the range typically reported for graphite-based screen-printed electrodes, where moderate disorder is expected due to the ink formulation and printing processes [33,38]. In addition, the D and G band positions listed in Table 1 are remarkably close for both electrodes, further supporting that the lab-fabricated ink reproduces the Raman characteristics of the commercial reference.

The in-plane crystallite size L_a_ was determined using the Tuinstra and Koenig equation [44,45], based on the D and G band intensity ratio obtained from the Raman spectral fitting (Table 2). This approach is widely applied to evaluate trends in the size of ordered sp^2^ domains when the same excitation wavelength and fitting procedure are used for all samples. The La values reported in Table 2 are consistent with the I_D_/I_G_ ratios, indicating similar domain sizes for SunTronic^®^ SPE and Graphite-SunTronic^®^ SPE, supporting the comparison between commercial and lab-fabricated inks.

Figure 1(c) shows a clear modification of the Graphite–SunTronic^®^ SPE after polishing with 2500-grit sandpaper followed by chemical treatment with 0.5 M H_2_SO_4_. Compared to the Graphite–SunTronic^®^ SPE, the D band exhibits a higher intensity than the G band, indicating an increased degree of structural disorder. The increase in ID/IG to 1.37 and decrease in La to 3.2 nm (Table 2) provide quantitative evidence for this observation. Raman investigations on graphene oxide and defect-engineered graphitic materials show that I_D_/I_G_ ratios above 1.0 indicate increased surface defects and edge sites [39,46]. Despite the increase in disorder-related contribution, the locations of the D, G, and 2D bands remain within the normal range of graphitic carbons (Table 1), demonstrating that graphitic domains are retained following treatment.

After PPy deposition, the Raman spectra show additional contributions in the low-wavenumber region, mainly for the Graphite–PPy composite prepared without CTAB, Figure 1(d). The bands located at approximately 450, 618, 927, 975, and 1059 cm^−1^ are compatible with vibrational modes of polypyrrole, including ring deformation and C–H/N–H-related vibrations [42,43]. For the PPy–CTAB-modified electrode, Figure 1(e), these polymer-related bands are less resolved because the spectrum is dominated by the carbon framework and by a broader spectral background produced after surfactant-assisted polymerization [43,44]. This behavior is consistent with a more dispersed and morphologically modified PPy-based layer, in which the polymer contribution appears as part of the overall carbon–polymer spectral response rather than as isolated sharp bands.

The comparison among Figure 1(c–e) shows that the main carbon-related D, G, and 2D bands remain visible after PPy and PPy–CTAB modification, indicating that the graphitic structure of the electrode is preserved during polymer deposition [45,46]. This observation is consistent with the I_D_/I_G_ values in Table 2, which decrease to 0.77 for Graphite-PPy composite prepared without CTAB polished with 2500 grit sandpaper and to 0.92 for Graphite-PPy composite SPE prepared with CTAB. Similar trends have been reported for polymer-modified carbon and graphene electrodes, where polymer deposition alters the surface composition without introducing significant additional disorder into the carbon structure [39,43]. This spectral evolution also agrees with the SEM–EDS analysis presented in the next subsection, where the modified electrodes exhibit a granular polymeric coating and elemental contributions associated with nitrogen-containing PPy structures.

The combined analysis of Figure 1 with Table 1 and Table 2 reveals a consistent evolution across the fabrication steps. The graphite–SunTronic^®^ carbon paste exhibits Raman characteristics comparable to commercial carbon inks. Mechanical polishing and acid treatment increase structural disorder, as indicated by the enhanced D band intensity and the higher $I_{D}/I_{G}$ ratio. After PPy-based modification, polymer-related spectral contributions are observed while the characteristic carbon Raman features are retained, indicating that the underlying graphitic framework remains structurally preserved. The differences between the PPy- and PPy–CTAB-modified electrodes are therefore associated mainly with changes in the carbon–polymer surface response and with the morphology induced by CTAB-assisted polymerization, rather than with the loss of the graphitic structure.

#### 3.1.2. SEM and EDS Characterizations

**Figure 2:**
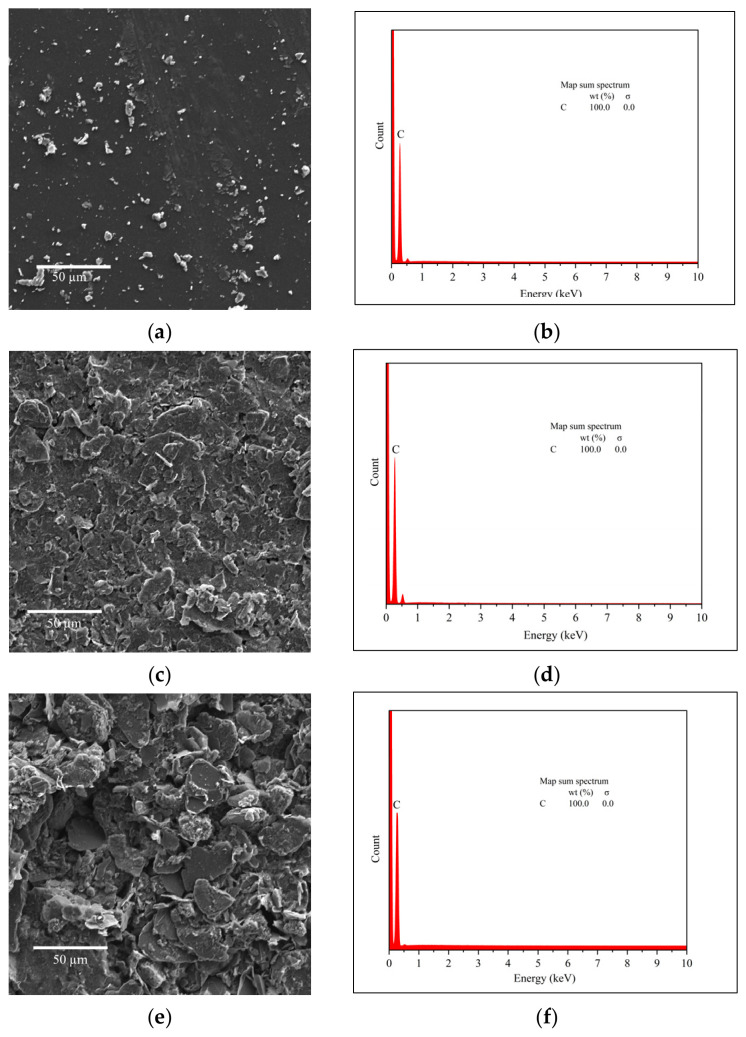

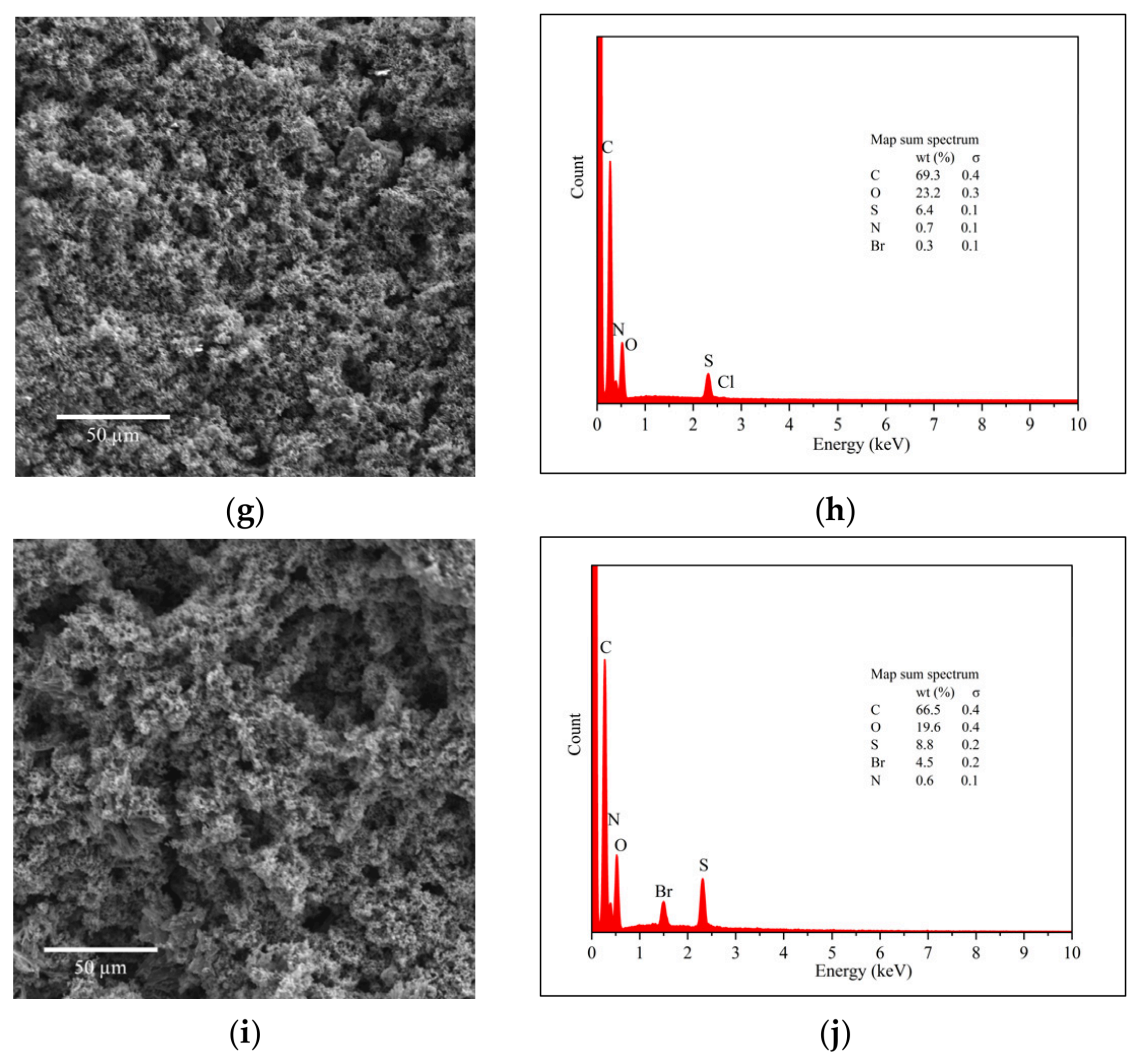
SEM and EDS spectrum, respectively, in: (**a**,**b**) SunTronic^®^ SPE, (**c**,**d**) Graphite-SunTronic^®^ SPE, (**e**,**f**) Graphite-SunTronic^®^ SPE polished with 2500 grit sandpaper and chemically treated with 0.5 M H_2_SO_4_, (**g**,**h**) Graphite-PPy Composite prepared without CTAB polished with 2500 grit sandpaper, (**i**,**j**) Graphite-PPy Composite SPE prepared with CTAB and polished with 2500 grit sandpaper.

Figure 2 shows the morphological and elemental evolution of the carbon electrode surfaces through the different fabrication and modification steps, complementing the Raman analysis by directly revealing the surface changes produced after PPy and PPy–CTAB incorporation. The SunTronic^®^ SPE exhibited a relatively smooth and compact surface with low roughness (Figure 2a), while its corresponding EDS spectrum confirms carbon as the main element, with no significant contribution from other elements (Figure 2b). In contrast, the Graphite-SunTronic^®^ SPE displays rougher and more heterogeneous surface morphology (Figure 2c), attributed to the lab-fabricated ink, although its elemental composition remains dominated by carbon, as confirmed by EDS (Figure 2d). After sanding and chemical cleaning with H_2_SO_4_ (Graphite-SunTronic^®^ SPE polished with 2500 grit sandpaper and chemically treated with 0.5 M H_2_SO_4_), a pronounced increase in surface roughness and porosity is observed, with greater exposure of carbon structures (Figure 2e), while the EDS spectrum still shows carbon as the predominant element, indicating that the treatment mainly promotes surface activation without introducing contaminants (Figure 2f).

Subsequent modification with PPy leads to more pronounced changes in both morphology and surface composition. The SPE-FAB-SA-PPy-CTAB0 surface presents a granular and highly rough morphology, consistent with the formation of a polymeric layer over the carbon substrate (Figure 2g). Its EDS spectrum shows the appearance of nitrogen-containing contributions associated with PPy, supporting the incorporation of the polymer phase on the electrode surface (Figure 2h). When CTAB is used during PPy synthesis, the SPE-FAB-SA-PPy-CTAB1 electrode exhibits a more uniform and densely nanostructured morphology (Figure 2i), indicating that the surfactant promotes a more organized polymer growth. The corresponding EDS spectrum also shows contributions from carbon, nitrogen, and elements related to the surfactant/dopant species, reinforcing the formation of a PPy–CTAB-modified interface (Figure 2j). Overall, the SEM–EDS results indicate that surface activation favors polymer deposition, while CTAB-assisted polymerization produces a more homogeneous carbon–polymer interface.

#### 3.1.3. Electrochemical Characterization

**Figure 3:**
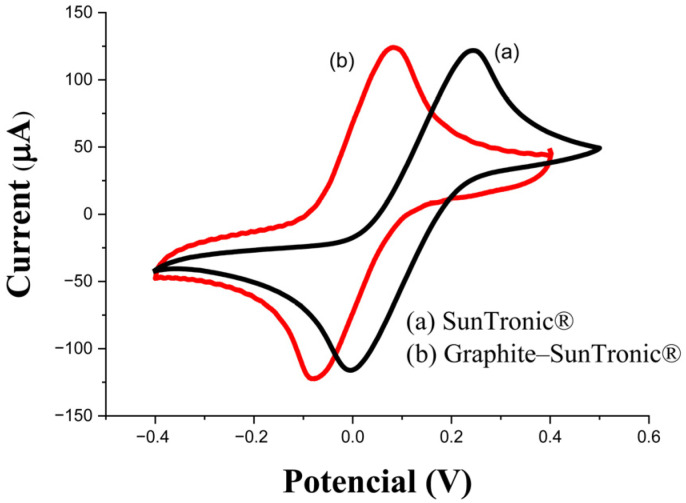
CV profiles of typical screen-printed electrodes prepared with (a) SunTronic^®^ ink (black) and (b) graphite–SunTronic^®^ composite ink (red). Measurements were performed in 5 mM K_3_Fe(CN)_6_/K_4_Fe(CN)_6_ prepared in 0.1 M KCl as redox probe at a scan rate of 50 mV s^−1^.

To enhance the electroactive properties of the SPE manufactured, the commercial flexographic carbon ink was blended with graphite powder previously dispersed in DMF to increase the effective surface area and charge storage at the electrode/electrolyte interface. The use of DMF played a critical role in this process by improving the homogeneous dispersion of graphite within the ink matrix, modulating the film-forming properties, and contributing to a more uniform and interconnected conductive network. This synergistic combination is expected to improve electron transport pathways while increasing the number of accessible active sites for double-layer formation. Consequently, the electrochemical response was markedly enhanced, as evidenced by the cyclic voltammetry (CV) profiles. The modified SPE displayed a higher current response and a more pronounced capacitive behavior, reflecting improved interfacial charge storage and overall electrochemical performance compared to the unmodified commercial carbon ink. The ratio of the anodic to cathodic peak (Ipa/Ipc) was nearly 1.0, indicating a faster electron transfer rate and confirming a more electrochemically reversible process. The lower $\Delta E_{p}$ observed for the graphite–SunTronic^®^ SPE indicates improved electron-transfer behavior under the ferri/ferrocyanide conditions used for electrode characterization (Figure 3).

The Graphite–SunTronic^®^ SPE, corresponding to curve (b) in Figure 3, exhibited reproducibility within the expected range for screen-printed carbon electrodes, demonstrating its suitability for comparative electrochemical analysis (Table 3). Cyclic voltammetry (CV) measurements performed on 30 independent electrodes yielded mean anodic peak current (Ipa) and cathodic peak current (Ipc) of 121.50 µA and 126.11 µA, with corresponding coefficients of variation of 7.95% and 8.24%, respectively. In addition, the peak potential separations (ΔEp) exhibited minimal variability, remaining below 1% for both anodic and cathodic peaks, indicating that electrical conductivity was maintained. This fact can be attributed to the effective dispersion and homogeneous distribution of graphite incorporated into the ink matrix.

### 3.2. Characterization of the PPy Film on the C-SPE

The electrochemical response of the PPy–CTAB composite was evaluated through the mass-specific capacitance obtained from the integrated area of the cyclic voltammograms, according to Equation (1) [24,47]

(1) $$C_{s}=\frac{\oint \left|i(V)\right|dV}{2mv∆V}$$

where $C_{s}$ is the mass-specific capacitance (F g^−1^), ∮∣i(V)∣ dV is the integrated absolute current response over the complete anodic and cathodic CV cycle (A V), m is the mass of active material deposited on the working electrode (g), ν is the scan rate (V s^−1^), and ΔV is the potential window width (V). Factor 2 accounts for the two potential sweeps in one complete CV cycle. All capacitance values were calculated within the potential range from −0.4 to +0.4 V, corresponding to ΔV=0.8 V.

**Figure 4:**
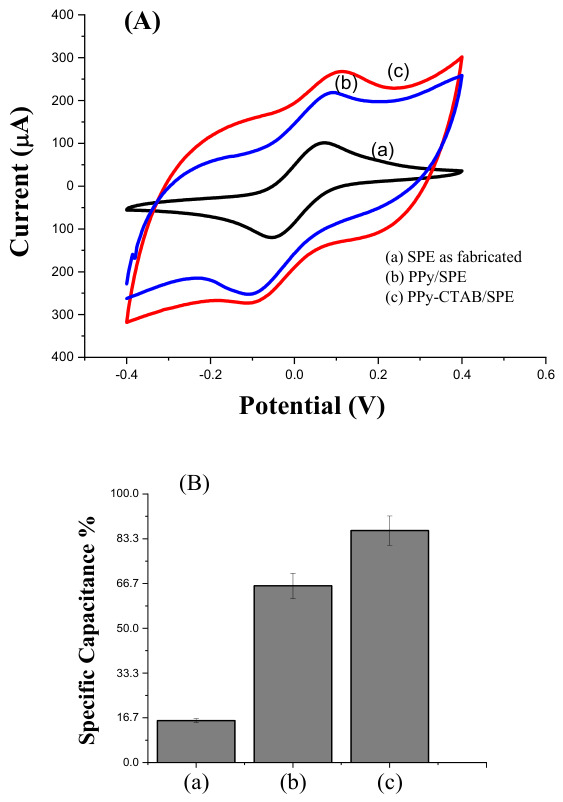
Typical SunTronic^®^-Graphite SPE: (a) As Fabricated, (b) PPy, (c) PPy-CTAB; in (**A**) CV profile obtained in presence of 5 mM of K_3_Fe(CN)_6_/K_4_Fe(CN)_6_ prepared in 0.1 M KCl as redox probe at 50 mV/s scan rate, and (**B**) Bar plot of Specific Capacitance for respective steps; bar error represents the standard deviation of three replicates.

Figure 4A compares the CV profiles of the as-fabricated SPE, PPy/SPE, and PPy–CTAB/SPE recorded under the same electrochemical conditions. The progressive increase in the voltammetric area after PPy deposition and CTAB-assisted PPy modification indicates an improvement in the charge-storage capability of the electrode. For Figure 4B, the capacitance values were expressed as normalized specific capacitance, calculated as $C_{s,norm}(\%)=(C_{s}/C_{s,ref})\times 100$, where $C_{s,ref}$ is the specific capacitance obtained for PPy–CTAB/SPE under the same experimental conditions. Thus, the PPy–CTAB/SPE response represents 100% in this comparison. For the stability assays, the normalized capacitance corresponds to capacitance retention, using the initial $C_{s}$ value measured before cycling or storage as 100%.

The PPy film resulted in an increase of approximately 4.28 times in the Cs as compared with the as-fabricated SPE, as can be seen in the bar graph (Figure 4B). In this synthesis, ammonium persulfate served as the oxidant for the in situ oxidative polymerization of pyrrole monomers, which preferentially occurred at surface adsorption sites, thereby enabling the formation of a uniform PPy coating. Moreover, strong π–π stacking interactions between the aromatic rings of PPy chains and the substrate planes further enhanced film homogeneity and adhesion [32,48]. Additionally, it is possible to produce films with higher porosity and to alter the pseudocapacitance and conductivity of the composite by adding an anionic charge. The use of CTAB in the PPy synthesis also resulted in an average increase of 30% in Cs (curve c) as compared to the PPy/SPE (curve b). This electrochemical gain is consistent with the Raman and SEM–EDS results, indicating that PPy–CTAB incorporation modified both the surface structure and the charge-storage properties of the electrode This enhancement is mainly attributed to the in situ oxidative polymerization of pyrrole monomers in a stable CTAB suspension, leading to the formation of PPy structures around CTAB micelles, which increases surface porosity, as confirmed by SEM. The resulting PPy–CTAB powder was subsequently dispersed in DMF under ultrasonication, which further promoted interactions between pyrrole monomers and the surface through π–π stacking, hydrogen bonding, and van der Waals forces. Consequently, a typical specific capacitance of 57.2 F g^−1^ with supercapacitive behavior was achieved via a simple one-step process, making this approach attractive for large-scale production compared to conventional in situ polymerization methods. Furthermore, biomolecular interactions can be monitored through direct capacitance measurements without the use of redox probes such as ferri/ferrocyanide, which is advantageous for point-of-care applications.

**Figure 5:**
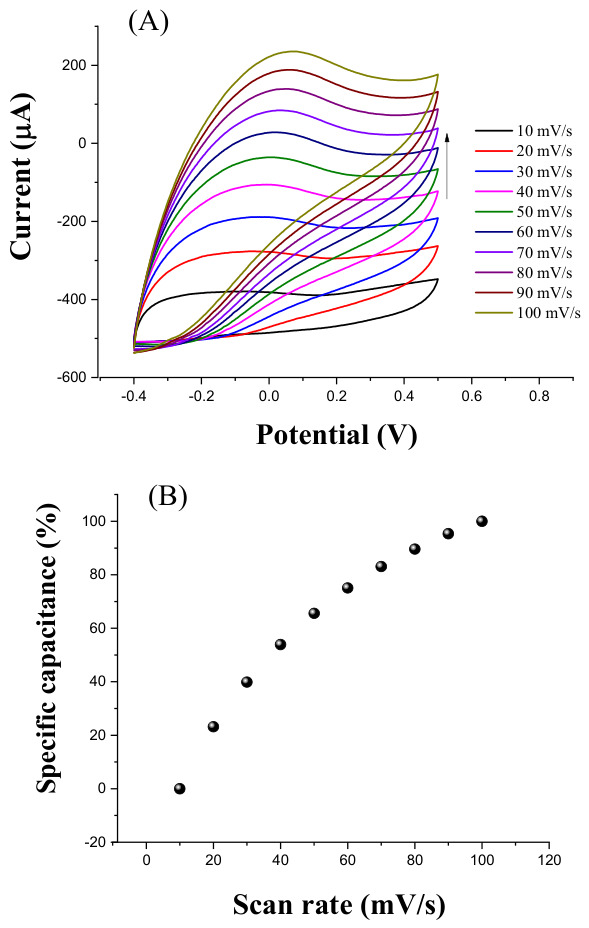
CV at different scan rates in (**A**); and effect of specific capacitance as function of scan rate (**B**). All measurements were performed in the presence of PBS.

**Figure 6:**
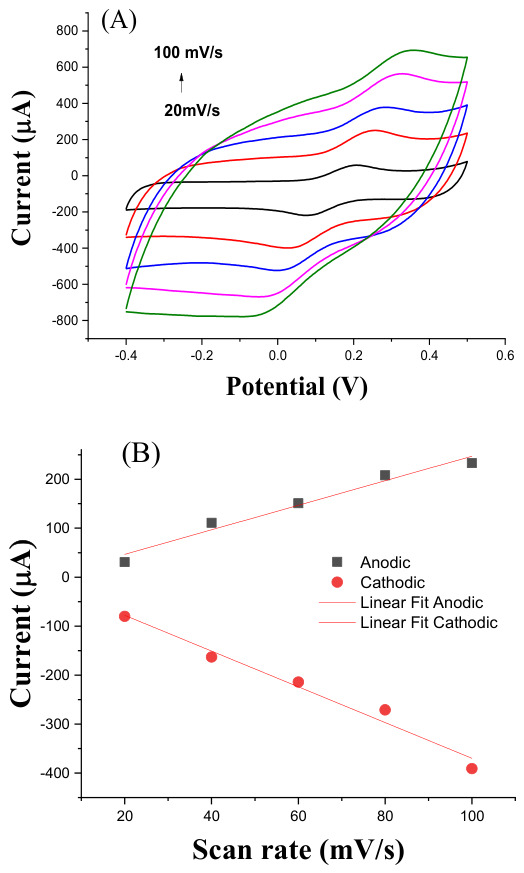
CV profile in different scan rates 20,40, 60, 80 and 100 mV/s of PPy-CTAB Graphite-SPE (**A**), and effect of scan rate on current of anodic and cathodic peaks (**B**). All measurements were performed in 5 mM of K_3_Fe(CN)_6_/K_4_Fe(CN)_6_ prepared at 0.1 M KCl.

Electrochemical characterization of the PPy–CTAB-modified electrodes was performed by CV in PBS, without soluble redox probes, to evaluate the intrinsic charge-storage behavior of the film (Figure 5A). The voltammetric profiles show a capacitive background with broad peak currents rather than an ideal quasi-rectangular shape. This response is consistent with the coexistence of electrical double-layer charging and pseudocapacitive redox processes of PPy, involving reversible doping/dedoping of the polymer backbone and ion exchange within the PPy–CTAB matrix [49,50]. The capacitance *Cs* increased with the scan rate (ν), reaching a plateau with an exponential behavior (Figure 5B), which can be attributed to limitations in the ion diffusion and restricted electrolyte access to inner active sites at higher scan rates. This fact suggests that, at lower ν, electrolyte ions can more effectively penetrate the porous structure of the electrode, maximizing the charge storage capacity, whereas at higher ν the response becomes increasingly surface-limited. These results indicated that the electrode modifications increase the electroactive surface area and improve the charge storage capability, while maintaining good ion transport properties, which are essential for sensitive electrochemical sensing applications (Figure 6B). The −0.4 to +0.4 V interval was used for the capacitance calculations because it corresponds to the stable operational window adopted for the analytical measurements, preserving the PPy–CTAB response while minimizing irreversible polarization effects in the polymer film [51].

The limitation of ionic diffusion in the presence of redox probe as a function of scan rate was analyzed by voltammograms (Figure 6A). The CTAB-PPy film under faradaic regime showed a capacitive behavior with a proportional increase in current response with scan rate, for both curves (i ∝ ν). It was observed that both the anodic and cathodic peaks showed a linear curve with R^2^ values of 0.969 and 0.975, respectively (*p* << 0.0), and coefficients of Pearson of 0.9847 and 0.9874, with a highly significant statistical correlation (*p* < 0.01), when applying the correlation hypothesis test (Figure 6B). These results demonstrate that charge diffusion on the electrode surface occurred, most likely due to the high porosity of the film, probably due to the CTAB doping during the synthesis.

The film stability of the PPy-CTAB film on the fabricated SPE was evaluated through 25 successive CVs performed in a potential window varying from−0.4 to 0.4 V at 50 mV/s of scan rate performed in 5 mM of K_3_Fe(CN)_6_/K_4_Fe(CN)^6^ prepared at 0.1 M KCl. According to Supplementary Figure S2, the changes on specific capacitance (Cs%) obtained from areas of voltammograms resulted in a coefficient of variation 5.78%, indicating a film with good stability. The PPy-CTAB film was also highly reproducible in the long term by evaluating six independent electrodes measured and a coefficient variation of 2.8% of Cs was found, which is much lower than reported in the literature for PPy films [17]. Measurements were performed in the presence of PBS containing 0.5% Tween 20 as electrolyte, under the same conditions to obtain the analytical response (Supplementary Figure S3).

The scan-rate-dependent CV response reflects the contribution of electroactive charge storage and ion transport within the PPy–CTAB-modified interface. The proportional increase in anodic and cathodic peak currents with scan rate indicates that the film maintains accessible electroactive sites and supports charge transport under the investigated conditions. This interfacial behavior provides the basis for using the PPy–CTAB/SPE platform to monitor changes produced during antibody immobilization and subsequent antigen recognition.

### 3.3. Immobilization and Analytical Responses to Spike-Protein

The immobilization of anti-Spike antibodies was carried out through non-covalent interactions, preserving their native conformation and favoring the exposure of Fab regions for subsequent interaction with the S protein. The incorporation of CTAB, a cationic surfactant, introduces quaternary ammonium moieties and long alkyl chains into the PPy-based film, creating interfacial domains that can favor antibody adsorption under physiological pH conditions. In this configuration, electrostatic contributions may arise from the cationic groups of CTAB, while hydrophobic interactions between the alkyl chains and hydrophobic regions of the protein can also contribute to antibody immobilization. The presence of CTAB induced morphological changes in the PPy matrix by increasing surface roughness and porosity, which enhanced the available surface area for biomolecule attachment. Thereby, the anti-Spike immobilization onto PPy-CTAB films predominantly occurred through physical adsorption mechanisms, including electrostatic and hydrophobic interactions. To block non-specific sites, 0.1% BSA was used, which also has an antifouling effect [18].

**Figure 7:**
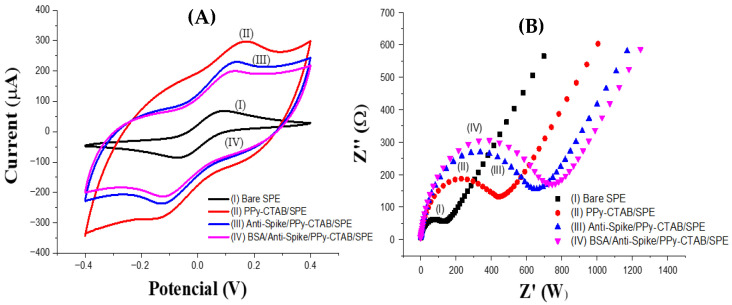
Electrochemical characterization of the stepwise immunosensor assembly. (**A**) Cyclic voltammograms and (**B**) Nyquist plots obtained for bare SPE, PPy–CTAB/SPE, anti-Spike/PPy–CTAB/SPE, and BSA/anti-Spike/PPy–CTAB/SPE in 5 mM K_3_[Fe(CN)_6_]/K_4_[Fe(CN)_6_] prepared in 0.1 M KCl. CV measurements were recorded at 50 mV s^−1^, while EIS was performed from 100 kHz to 0.1 Hz using an AC amplitude of 100 mV at 0.224 V. The electrochemical changes support the sequential formation of the PPy–CTAB-based biorecognition interface.

The stepwise assembly of the immunosensor was monitored by CV and EIS after each modification stage, as shown in Figure 7A,B. In this analysis, ferri/ferrocyanide was used as an interfacial redox probe to track the surface evolution during PPy–CTAB deposition, anti-Spike immobilization, and BSA blocking. In Figure 7A, PPy–CTAB deposition increased the voltammetric response compared with the bare SPE, reflecting the enhanced charge-storage capability and electroactive contribution of the polymer-modified interface. The subsequent immobilization of anti-Spike and blocking with BSA decreased the current response and voltammetric area, consistent with the formation of less-conductive biomolecular layers on the PPy–CTAB/SPE surface, thereby partially hindering charge transfer and attenuating the capacitive response of the film [20]. The Nyquist plots in Figure 7B showed complementary changes in the impedance response after each modification step, further supporting the sequential formation of the PPy–CTAB-based biorecognition interface.

**Figure 8:**
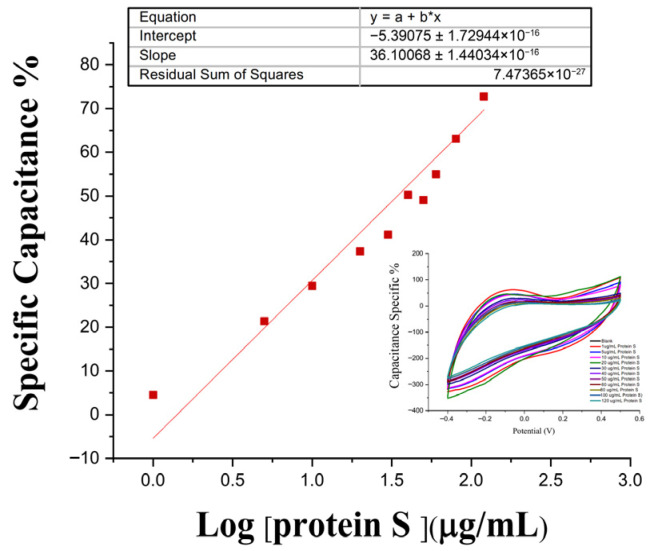
Analytical curve obtained of specific capacitance as a function of concentration of Protein S. Inset: CVs profiles obtained in response to protein S in the presence of PBS-Tween at 50 mV/s scan rate.

Analytical curves were obtained by incubating the produced immunosensor with an aliquot (25 µL) of Protein S samples prepared in PBS-Tween. After each incubation, the electrode was rinsed gently, stirring for 2 min in a beaker, and the electrochemical signals were recorded. According to Figure 8, increasing concentrations of Spike protein led to a progressive decrease in the voltammetric area, reflecting a reduction in specific capacitance (C_s_), likely due to the formation of an insulating antigen layer at the electrode interface. This procedure was repeated continuously, up to the maximum concentration tested, 100 µg of spike protein. A linear range was observed between 1.0 µg/mL and 100 µg Spike protein (R^2^ = 0.998, *p* < 0.05), with a limit of detection (LOD) of 0.45 µg/mL. The limit of detection (LOD) was estimated using the 3σ/S approach, where σ corresponds to the standard deviation of the blank measurements and S is the sensitivity of the sensor, defined as the slope of the calibration curve.

The S protein is a highly specific biomarker of COVID-19, which has driven extensive efforts toward the development of biosensing platforms. A comparative analysis of reported electrochemical immunosensors reveals that, although this present work did not achieve an ultrasensitive LOD it offers significant practical engineering advantages that are critical for real-world applications (Table 4). For comparison, previously reported electrochemical immunosensors based on Cu_2_O nanocubes, electrodeposited gold nanostructures, and SPE/Au impedimetric interfaces have commonly used EIS and soluble redox mediators for signal generation [52,53]. However, soluble redox probes may compromise antibody activity, promote nonspecific adsorption onto the sensor surface, induce baseline instability, and limit their applicability in rapid point-of-care settings [54]. A magnetic bead/carbon-black platform has also been reported for SARS-CoV-2 S and N protein detection in Saliva, but this strategy used a labeled immunosensor format involving secondary antibodies and magnetic beads, increasing assay complexity and operation time [55]. In contrast, the present platform is based on a probe-free strategy and a simplified fabrication process, avoiding the electropolymerization process and complex nanostructured steps. These features enable rapid, low-cost, and scalable production, which is particularly advantageous in pandemic scenarios, where accessible and deployable diagnostic solutions are urgently required. Although the detection limit is higher, it remains within a clinically relevant range, comparable to that used at several commercially available diagnostic tests, reinforcing the practical applicability of the proposed approach.

**Figure 9:**
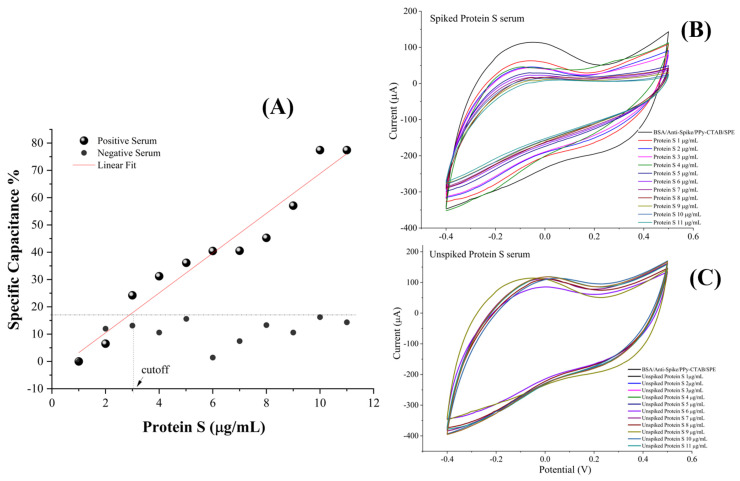
Analytical responses of specific capacitance in the study of specificity against Spiked and Unspiked Protein S pooled samples and linear Fit (**A**). CV profiles in response to successive incubation with spiked (**B**) and unspiked (**C**) protein S of COVID-19. Measurements obtained in the presence of PBS-Tween at 50 mV/s scan rate.

Analytical responses to S protein of SARS-CoV-2 were also measured in spiked and unspiked in complex samples. It used human serum to evaluate the capacity of this immunosensor to differentiate antigen–antibody affinity, characterizing the diagnostical specificity by the cut-off. According to CVs generated in the presence of PBS tween-20, the Capacitance specific increased with the concentration of protein S, unlike unspiked samples, showed an irregular response as expected (Figure 9).

The cut-off value represents the level of reaction discriminant and was obtained as the means of Cs of unspiked (blank) serum responses of 10 sample values plus 20%, which corresponds to the calculation according to the standards used in clinical laboratories. Herein, a cut-off of approximately 2.3 μg/mL of S protein was estimated. According to clinical practice, these values found allow the diagnosis of COVID-19, being of clinical relevance.

## 4. Conclusions

A capacitive immunosensor for COVID-19 was developed, based on direct measurements without the use of any redox probe for analytical responses. The PBS 0.05% Tween was used in the measurements, which allows a platform immunosensor to be simpler, more practical, and faster technology without the release of toxic substances. The use of conductive polymers like the PPy with CTAB doped to improve its conductive characteristics, in which the sensing surface may generate differences in electrical potentials in their gaps, resulting in electrochemical surfaces with a high pseudocapacitance and capable of generating quantum phenomena, and the possibility of faradaic measurements and direct monitoring of antigen–antibody interactions. In this context, the proposed film on the sensing platform exhibits electrochemically stable redox activity, which makes the platform easy to synthesize and capable of mass production, making it an imperative strategy for the massive monitoring of a disease like the COVID-19.

## Figures and Tables

**Table 1:** Results of the Raman spectra according to SPE prepared with (a) SunTronic^®^, (b) graphite–SunTronic^®^, (c) polished/acid-treated graphite–SunTronic^®^, (d) polished/acid-treated graphite–PPy (without CTAB), and (e) polished/acid-treated graphite–PPy (with CTAB).

References	(a)	(b)	(c)	(d)	(e)
γ N–H [42]				450	
γ ring [42]				618	
γ C–H [42,43]				927	
δ ring [42,43]				975	
δ ring, C–H [42]				1059	
D(1227) [39]	1227	1158	1200	1255	1257
D (1350) [33,38]	1347	1347	1347	1353	1361
D” (1512) [38]	1461	1500	1508	1489	1469
G (1585) [33,38]	1575	1582	1583	1584	1555
D’ (1620) [33]	1741	1619	1616	1622	1608
2D (2700) [33]	2683	2711	2702	2707	2728
D + D’ [39]	2886	2951	2936	2956	2888

**Table 2:** Relative intensity for some Raman peaks of SPE prepared with: (a) SunTronic^®^, (b) graphite—SunTronic^®^, (c) polished/acid-treated graphite—SunTronic^®^, (d) polished/acid-treated graphite—PPy (without CTAB), and (e) polished/acid-treated graphite—PPy (with CTAB).

Description	I_D_	I_G_	I_D_/I_G_	*L_a_* (nm)
SunTronic^®^ SPE	55	79	0.70	6.3
Graphite-SunTronic^®^ SPE	57	73	0.78	5.6
Graphite-SunTronic^®^ SPE polished with 2500 grit sandpaper and chemically treated with 0.5 M H_2_SO_4_	82	60	1.37	3.2
PPy prepared without CTAB on graphite-SunTronic^®^ SPE polished with 2500 grit sandpaper	58	75	0.77	5.7
PPy-CTAB on graphite-SunTronic^®^ SPE and polished with 2500 grit sandpaper	59	64	0.92	4.8

**Table 3:** Reproducibility of graphite-SunTronic^®^-SPE: Average and coefficient of variation in the electrochemical parameters extracted from CVs of 30 different electrodes.

	Ipa (µA)	Ipc (µA)	Ipa/Ipc	Epa (V)	Epc (V)	ΔEP (V)
Average	121.50	−126.11	−0.96	0.07	−0.06	0.13
Coefficient of variation%	7.95	8.24	0.36	0.48	1.37	0.65

Ipa: anodic peak current; Ipc: cathodic peak current; Epa: anodic peak potential; Epc: cathodic peak potential; ΔEp: peak-to-peak separation calculated as $E_{pa}-E_{pc}$.

**Table 4:** Analytical performance comparison of antibody-based electrochemical immunosensors for SARS-CoV-2 Spike protein detection.

Sensor Platform	Material	Technique	LOD	Linear Range	Reference
Cu_2_O nanocube-based immunosensor	Cu_2_O Nanocube/GCE	EIS	**0.04 fg/mL**	0.25 fg/mL to 1 μg/mL	[52]
Gold Nanoparticle-based immunosensor	Gold Electrode/SPE	EIS	**0.25 ng/mL**	0.5–100 ng/mL	[56]
Magnetic beads-labeled immunosensor	Carbon-black electrode	SWV	**19 ng/mL^−1^**	16 ng/mL–2.5 µg/mL	[55]
Impedimetric immunosensor	SPE/Au	EIS	**25 ng/mL**	10 to 100 ng/mL	[53]
This work	PPy–CTAB/SPE	Capacitance	**0.45 µg/mL**	1 to 100 **µg/mL**	This work

## Data Availability

Please add the corresponding content of this part.

## Supplementary Materials

The following supporting information can be downloaded at: https://www.mdpi.com/article/10.3390/mi17060731/s1.

## Abbreviations

Nomenclature	Name
ST-SPE	SunTronic^®^ SPE
G-SPE	Graphite–SunTronic^®^ SPE
aG-SPE	Graphite–SunTronic^®^ SPE polished with 2500 grit sandpaper and chemically treated with 0.5 M H_2_SO_4_
PPy/G-SPE	Graphite–PPy composite prepared without CTAB
PPy–CTAB/G-SPE	Graphite–PPy composite prepared with CTAB/PPy–CTAB on graphite–SunTronic^®^ SPE
Ab/PPy–CTAB/G-SPE	Anti-Spike immobilized on PPy–CTAB/SPE
BSA/Ab/PPy–CTAB/G-SPE	BSA-blocked anti-Spike/PPy–CTAB/SPE
