# Supplementary material for: A Capacitive Immunosensor Based on a Polypyrrole–CTAB for Probe-Free Detection of SARS-CoV-2 Spike Protein
Source: Micromachines (Basel). 2026 Jun 17;17(6):731. doi: 10.3390/mi17060731 (PMC13303280; doi:10.3390/mi17060731)
Supplement: Supplementary file 1 [file micromachines-17-00731-s001.zip › micromachines-4334988-supplementary.pdf]

Figure S1. Schematic process for PPy-CTAB film preparation and modifying the Graphite-SunTronic SPE for analytical measurements.

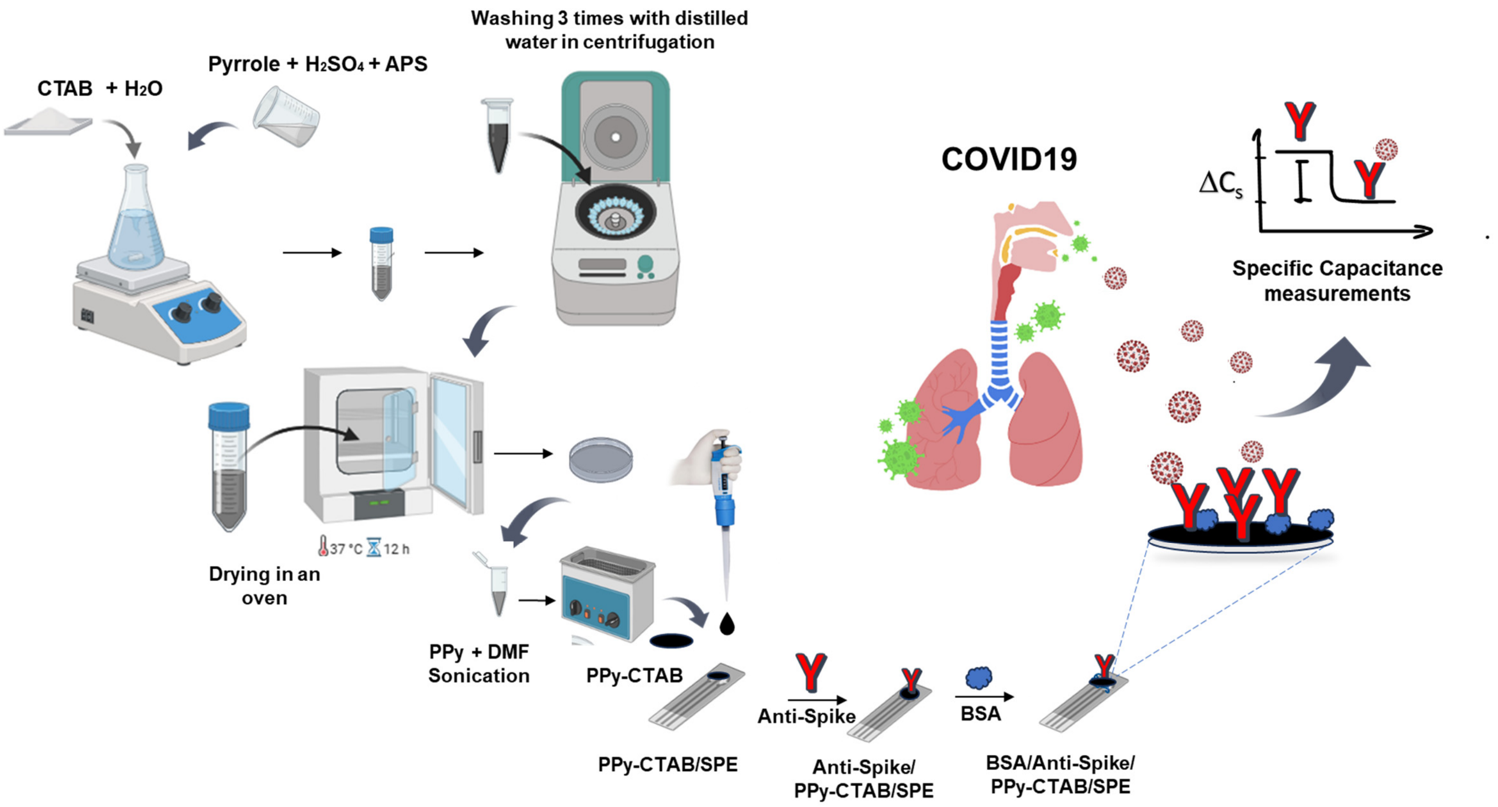

Figure S2. CV profiles of PPy-CTAB on fabricated SPE, in 25 successive cycles performed in 5mM of $K_3Fe(CN)_6/K_4Fe(CN)^6$ prepared at 0.1 M KCl. Figure S2.

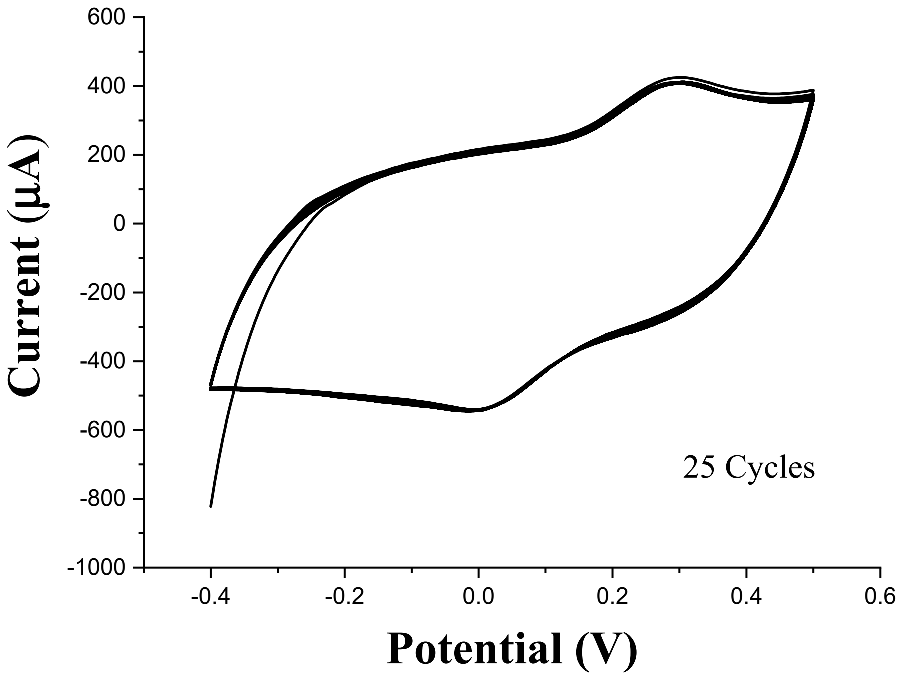

Figure S3. CV profiles of PPy-CTAB obtained in the presence of PBS containing 0.5% Tween 20 from six different electrodes, in (A). All measurements were performed at 50 mV/s of scan rate.

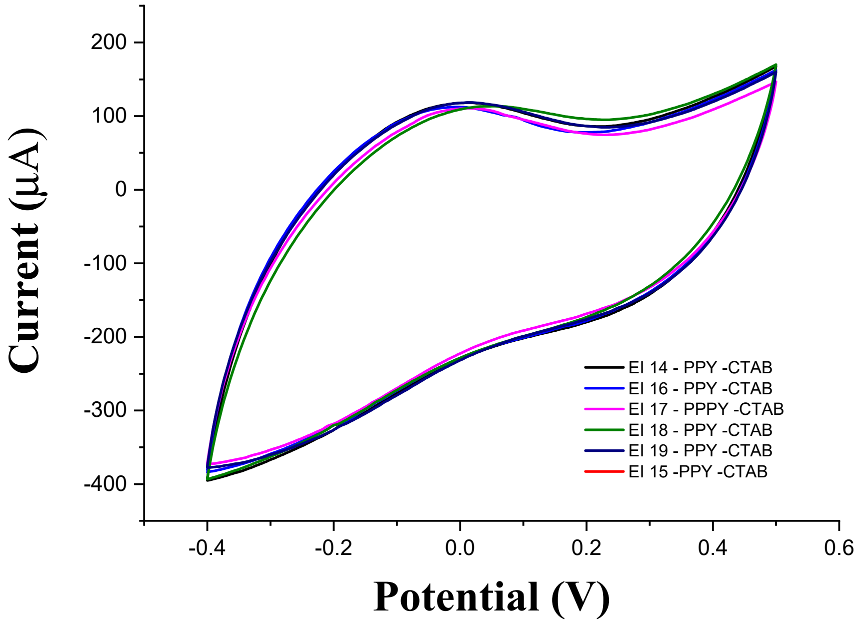

ANNEX - REFINEMENT

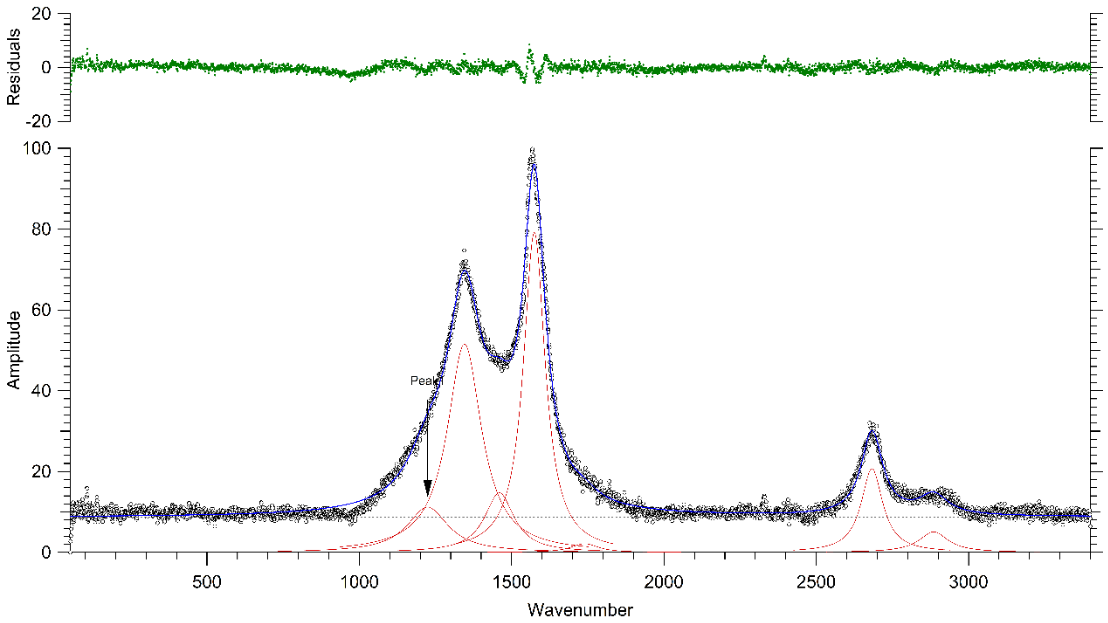

SPE-COM

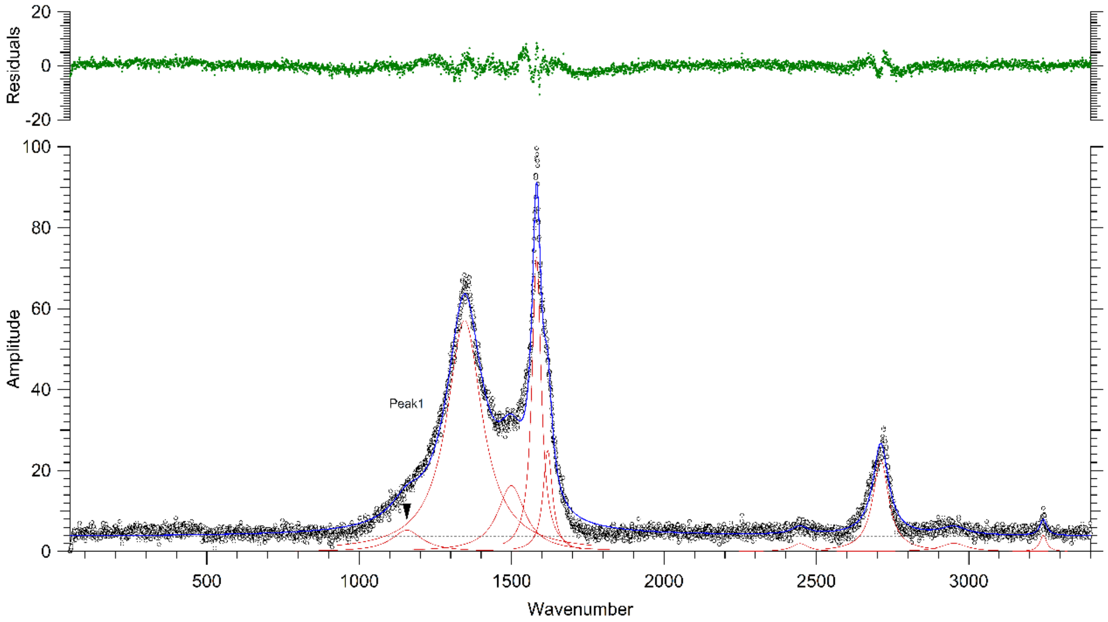

Residuals
20
0
-20
Amplitude
100
80
60
40
20
0
Peak1
500
1000
1500
2000
2500
3000
Wavenumber

SPE-FAB

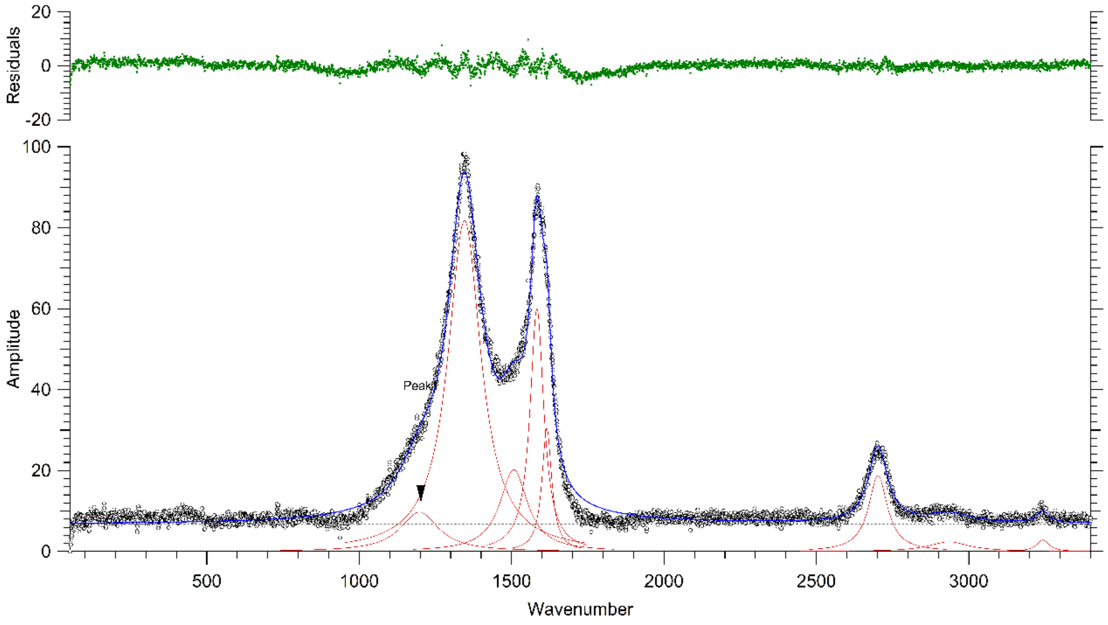
20
0
-20
Residuals
100
80
60
40
20
0
Amplitude
Peak
500
1000
1500
2000
2500
3000
Wavenumber

SPE-FAB-SA

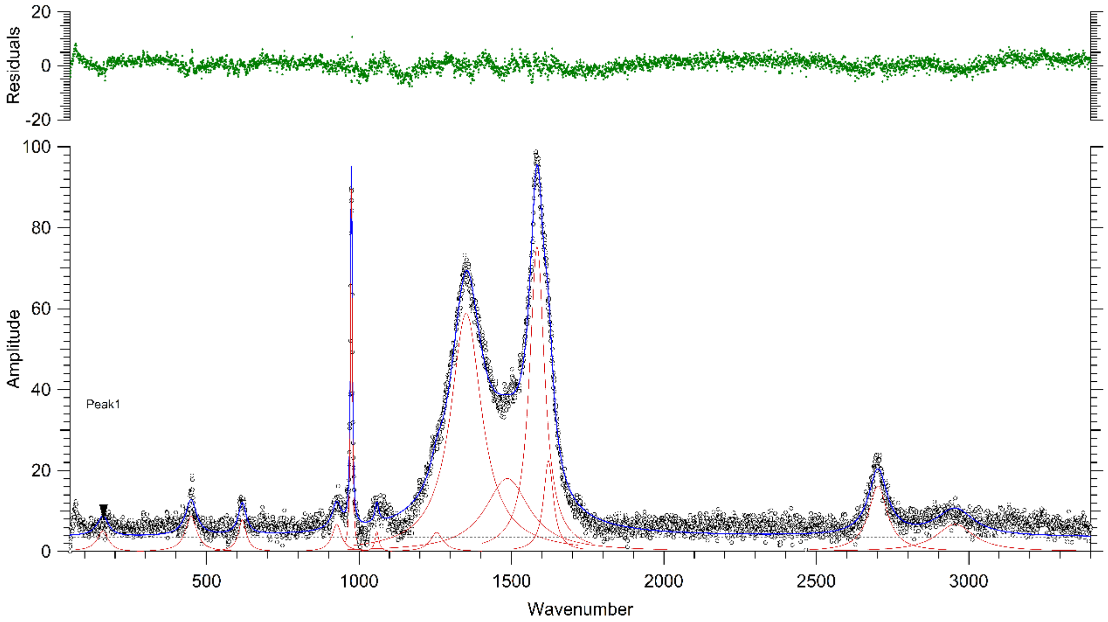

SPE-FAB-SA-PPy-CTAB0

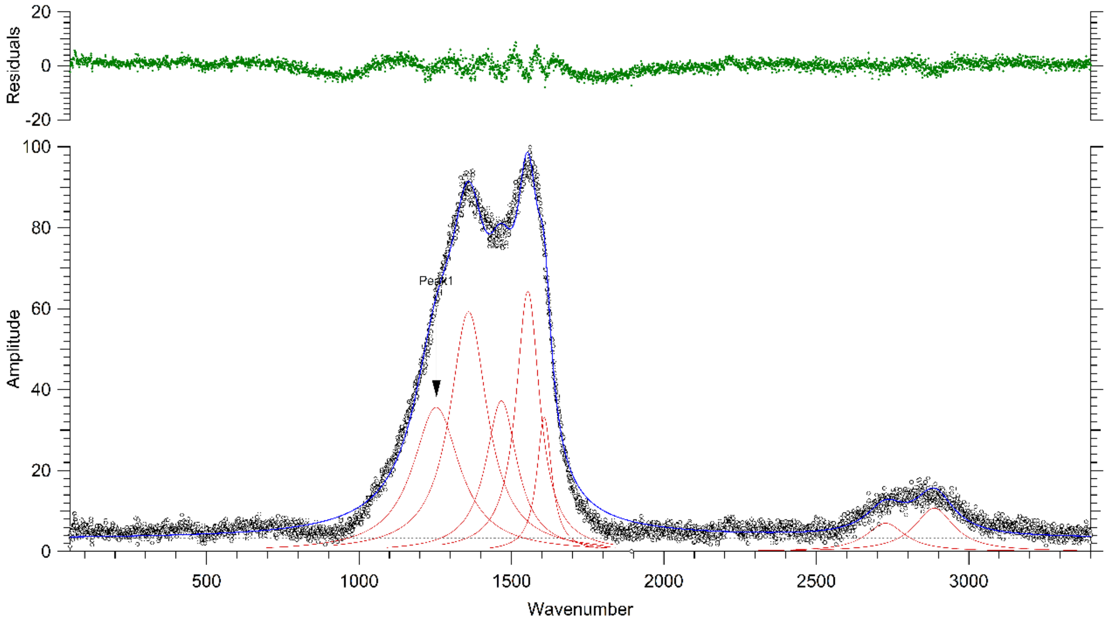

SPE-FAB-SA-PPy-CTAB1
